# A hyperaldosteronism subtypes predictive model using ensemble learning

**DOI:** 10.1038/s41598-023-29653-2

**Published:** 2023-02-21

**Authors:** Shigehiro Karashima, Masaki Kawakami, Hidetaka Nambo, Mitsuhiro Kometani, Isao Kurihara, Takamasa Ichijo, Takuyuki Katabami, Mika Tsuiki, Norio Wada, Kenji Oki, Yoshihiro Ogawa, Ryuji Okamoto, Kouichi Tamura, Nobuya Inagaki, Takanobu Yoshimoto, Hiroki Kobayashi, Miki Kakutani, Megumi Fujita, Shoichiro Izawa, Tetsuya Suwa, Kohei Kamemura, Masanobu Yamada, Akiyo Tanabe, Mitsuhide Naruse, Takashi Yoneda, JPAS/JRAS Study Group, Shigehiro Karashima, Mitsuhiro Kometani, Isao Kurihara, Takamasa Ichijo, Takuyuki Katabami, Mika Tsuiki, Norio Wada, Kenji Oki, Yoshihiro Ogawa, Ryuji Okamoto, Kouichi Tamura, Nobuya Inagaki, Takanobu Yoshimoto, Hiroki Kobayashi, Miki Kakutani, Megumi Fujita, Shoichiro Izawa, Tetsuya Suwa, Kohei Kamemura, Masanobu Yamada, Akiyo Tanabe, Mitsuhide Naruse, Takashi Yoneda, Hiroshi Ito, Yoshiyu Takeda, Hiromi Rakugi, Koichi Yamamoto, Masayoshi Soma, Toshihiko Yanase, Hisashi Fukuda, Shigeatsu Hashimoto, Yuichi Ohno, Katsutoshi Takahashi, Hirotaka Shibata, Yuichi Fujii, Tomoko Suzuki, Atsushi Ogo, Ryuichi Sakamoto, Tatsuya Kai, Tomikazu Fukuoka, Shozo Miyauchi

**Affiliations:** 1 Institute of Liberal Arts and Science, Kanazawa University, Kanazawa, Japan; 2 School of Electrical Information Communication Engineering, College of Science and Engineering, Kanazawa University, Kanazawa, Japan; 3 Department of Endocrinology and Metabolism, Kanazawa University Graduate School of Medicine, Kanazawa, Japan; 4 Department of Medical Education, National Defense Medical College, Tokorozawa, Japan; 5 Department of Endocrinology, Metabolism and Nephrology, Keio University School of Medicine, Tokyo, Japan; 6 Department of Diabetes and Endocrinology, Saiseikai Yokohamashi Tobu Hospital, Yokohama, Japan; 7 Division of Metabolism and Endocrinology, Department of Internal Medicine, St. Marianna University Yokohama City Seibu Hospital, Yokohama, Japan; 8 Department of Endocrinology and Metabolism, National Hospital Organization Kyoto Medical Center, Kyoto, Japan; 9 Department of Diabetes and Endocrinology, Sapporo City General Hospital, Sapporo, Japan; 10 Department of Molecular and Internal Medicine, Graduate School of Biomedical and Health Sciences, Hiroshima University, Hiroshima, Japan; 11 Department of Medicine and Bioregulatory Science, Graduate School of Medical Sciences, Kyushu University, Fukuoka, Japan; 12 Department of Cardiology and Nephrology, Mie University Graduate School of Medicine, Tsu, Japan; 13 Department of Medical Science and Cardiorenal Medicine, Yokohama City University Graduate School of Medicine, Yokohama, Japan; 14 Division of Nephrology and Hypertension, Yokohama City University Medical Center, Yokohama, Japan; 15 Department of Diabetes, Endocrinology, and Nutrition, Kyoto University, Kyoto, Japan; 16 Department of Molecular Endocrinology and Metabolism, Tokyo Medical and Dental University, Tokyo, Japan; 17 Division of Nephrology, Hypertension, and Endocrinology, Nihon University School of Medicine, Tokyo, Japan; 18 Division of Diabetes, Endocrinology, and Clinical Immunology, Department of Internal Medicine, Hyogo College of Medicine, Hyogo, Japan; 19 Division of Nephrology and Endocrinology, The University of Tokyo, Tokyo, Japan; 20 Division of Endocrinology and Metabolism, Faculty of Medicine, Tottori University, Yonago, Japan; 21 Department of Diabetes and Endocrinology, Graduate School of Medicine, Gifu University, Gifu, Japan; 22 Department of Cardiology, Shinko Hospital, Hyogo, Japan; 23 Department of Medicine and Molecular Science, Gunma University Graduate School of Medicine, Maebashi, 371-8511 Japan; 24 Division of Endocrinology, National Center for Global Health and Medicine, Tokyo, Japan; 25 Endocrine Center, Ijinkai Takeda General Hospital, Kyoto, Japan; 26 Department of Health Promotion and Medicine of the Future, Kanazawa University Graduate School of Medicine, Kanazawa, Japan; 27 Faculty of Transdisciplinary Sciences, Institute of Transdisciplinary Sciences, Kanazawa University, 13-1, Takara-Machi, Kanazawa, 920-8641 Japan; 28 Division of Endocrinology and Hypertension, Department of Internal Medicine, Kanazawa University Graduate School of Medicine, Kanazawa, Japan; 29 Department of Geriatric and General Medicine, Osaka University Graduate School of Medicine, Suita, Japan; 30 Division of General Medicine, Department of Internal Medicine, Nihon University School of Medicine, Tokyo, Japan; 31 Department of Endocrinology and Diabetes Mellitus, Faculty of Medicine, Fukuoka University, Fukuoka, Japan; 32 Division of Nephrology, Hypertension, Endocrinology, and Diabetology/Metabolism, Fukushima Medical University Hospital, Fukushima, Japan; 33 Department of Metabolism, Showa General Hospital, Kodaira, Japan; 34 Department of Endocrinology, Metabolism, Rheumatology and Nephrology, Faculty of Medicine, Oita University, Yufu, Japan; 35 Department of Cardiology, JR Hiroshima Hospital, Hiroshima, Japan; 36 Department of Public Health, School of Medicine, International University of Health and Welfare, Otawara, Japan; 37 Department of Endocrinology and Metabolism, Clinical Research Institute, National Hospital Organization, Kyushu Medical Center Kyushu Medical Center, Fukuoka, Japan; 38 Department of Internal Medicine, Sakura Hospital, Sayama, Japan; 39 Internal Medicine, Matsuyama Red-Cross Hospital, Matsuyama, Japan; 40 Uwajima City Hospital, Uwajima, Japan

**Keywords:** Endocrinology, Health care, Medical research, Oncology

## Abstract

This study aimed to develop a machine-learning algorithm to diagnose aldosterone-producing adenoma (APA) for predicting APA probabilities. A retrospective cross-sectional analysis of the Japan Rare/Intractable Adrenal Diseases Study dataset was performed using the nationwide PA registry in Japan comprised of 41 centers. Patients treated between January 2006 and December 2019 were included. Forty-six features at screening and 13 features at confirmatory test were used for model development to calculate APA probability. Seven machine-learning programs were combined to develop the ensemble-learning model (ELM), which was externally validated. The strongest predictive factors for APA were serum potassium (s-K) at first visit, s-K after medication, plasma aldosterone concentration, aldosterone-to-renin ratio, and potassium supplementation dose. The average performance of the screening model had an AUC of 0.899; the confirmatory test model had an AUC of 0.913. In the external validation, the AUC was 0.964 in the screening model using an APA probability of 0.17. The clinical findings at screening predicted the diagnosis of APA with high accuracy. This novel algorithm can support the PA practice in primary care settings and prevent potentially curable APA patients from falling outside the PA diagnostic flowchart.

## Introduction

Primary aldosteronism (PA), with a reported prevalence of 5–15% and 2.6–12.7% in hypertensive^1–4^ and primary care patients, respectively, is a common cause of secondary hypertension^5,6^. PA, compared with primary hypertension, confers an increased risk of cardiovascular and cerebrovascular diseases, diabetes, and metabolic syndrome^7–9^. Patients with unilateral PA, mainly aldosterone-producing adenoma (APA), can be surgically cured with unilateral adrenalectomy. PA is commonly caused by APA, unilateral or bilateral adrenal hyperplasia (BAH), or in rare cases, adrenal carcinoma or inherited conditions of familial hyperaldosteronism^4^.

Diagnosing the PA subtype necessitates complex processes, such as screening, confirmatory testing, imaging examination (including computed tomography [CT]), and adrenal venous sampling (AVS)^4,10,11^. Detailed examinations other than screening tests are performed at hospitals and clinics specializing in endocrine hypertension. However, 50–70% of PA patients are diagnosed with bilateral PA^12,13^, which is treated with medication. A more efficient diagnosis of APA is needed.

Recently, machine-learning algorithms (MLAs) have generated considerable interest in developing clinical informatics tools for disease diagnosis, staging, and prognosis^14,15^. Several MLAs, such as the supervised learning models Random Forest (RF) and multiple-layer perceptron (MLP), have been widely used in classification and regression analyses^16,17^. These supervised learning models apply data-mining techniques to recognize hidden patterns in complex data for better prediction of clinical outcomes than traditional statistical models, especially in large-scale datasets. Particularly, ensemble methods are a state-of-the-art solution that train multiple machine learning models and combine their prediction results. An ensemble method provides better predictive performance than a single machine learning model^18,19^.

Several PA laterality prediction models exist^20–23^, but few have been tested using artificial intelligence. Moreover, many of these models were developed using findings from CT and confirmatory tests instead of focusing on clinical data obtained by general practitioners. The ability to predict PA subtypes using primary care data would help ensure that no APA cases are missed that should be diligently examined according to the PA diagnostic flowchart. Such a predictive model may also provide economic benefits to patients with BAH by reducing the cost of CT scans and AVS, which are unnecessary in retrospect. The purpose of this study was to develop and validate an ensemble learning model to predict APA probability based on clinical characteristics of a nationwide cohort database in Japan.

## Results

### Patient characteristics

A total of 4057 patients with PA were registered in the Japan Rare/Intractable Adrenal Diseases Study (JRAS) database. We defined APA in three ways. First, APA Set A was defined as lateral aldosterone production in the adrenal vein and detection of an adrenal adenoma on pathology. Second, APA Set B was defined as APA with complete or partial biochemical improvement after surgery in addition to the conditions of Set A. APA Set C was defined as PA with lateral aldosterone production in the adrenal vein and complete or partial biochemical improvement after surgery. In Set C, pathology results were not included in the criteria. Based on the inclusion and exclusion criteria, 545 and 1352 patients with APA Set A and BAH, respectively, were included in this study (Supplementary Fig. S1). Table 1 shows the baseline characteristics of the patients with APA Set A and BAH. Supplementary Table S1 compares the clinical background of the APA Set B or Set C patient groups with BAH.

**Table 1:** Comparison of baseline characteristics of patients with APA and BAH.

Parameters	APA SET_A (n = 545)	BAH (n = 1352)	P-value
Age (years)	50 (40–59)	52 (45–61)	< 0.001
Sex, female (%)	50.5	54.4	0.12
BMI (kg/m^2^)	23.7 (20.9–26.6)	24.7 (22.3–27.5)	< 0.001
Age of onset of hypertension (years)	39 (34–48)	45 (38–52)	< 0.001
Family history of hypertension (%)	60.7	63.1	0.72
Current or past smoking (%)	36.9	33.5	0.31
Current or past drinking (%)	54.1	52.0	0.87
SBP at the first visit (mmHg)	160 (148–180)	152 (140–170)	< 0.001
DBP at the first visit (mmHg)	100 (88–110)	98 (87–106)	< 0.001
SBP with antihypertensive treatment (mmHg)	141 (130–154)	140 (130–150)	0.42
DBP with antihypertensive treatment (mmHg)	87 (78–96)	88 (78–96)	0.75
Biochemistry			
BUN (mg/dL)	13.0 (10.3–15.3)	13.0 (11.0–15.5)	0.22
Serum creatinine (mg/dL)	0.70 (0.59–0.88)	0.70 (0.60–0.82)	0.51
eGFR (mL/min per1.73 m^2^)	79.8 (65.5–94.1)	78.3 (67.2–90.3)	0.28
s-UA (mg/dL)	5.0 (4.2–6.1)	5.3 (4.5–6.3)	0.002
s-K at the first visit (mEq/L)	2.9 (2.5–3.2)	3.4 (3.2–3.6)	< 0.001
s-K with supplement (mEq/L)	3.3 (2.9–3.6)	3.9 (3.7–4.1)	< 0.001
s-Na (mEq/L)	143 (141–144)	141 (140–143)	< 0.001
s-Cl (mEq/L)	105 (103–106)	105 (104–107)	< 0.001
FBS (mg/mL)	96 (89–107)	98 (92–108)	0.004
HbA1c (NGSP) (%)	5.5 (5.2–5.9)	5.7 (5.4–6.0)	< 0.001
TC, total cholesterol (mg/dL)	188 (167–207)	195 (172–219)	0.06
TG, triglyceride (mg/dL)	93 (68–139)	112 (80–159)	< 0.001
LDL, LDL-cholesterol (mg/dL)	109 (92–130)	115 (95–135)	0.03
HDL, HDL-cholesterol (mg/dL)	55 (46–67)	53 (44–65)	0.11
Urine test			
Urine protein (n, %)			
(−) or (±)	408 (83.6)	1116 (92.3)	
(+)	50 (10.2)	67 (5.5)	
(2 +)	14 (2.9)	14 (1.2)	
(3 +)	16 (3.3)	12 (1.0)	
PA complications			
Stroke (%)	6.2	4.1	0.04
IHD (%)	2.2	1.7	0.46
Heart failure (%)	0.73	0.22	0.18
Atrial fibrillation (%)	0.92	1.6	0.28
CKD (%)	4.6	2.9	0.08
Hyperuricemia (%)	4.8	3.4	0.16
Diabetes (%)	13.0	13.6	0.70
Dyslipidemia (%)	22.4	26.9	0.04
SAS (%)	3.5	3.1	0.67
Depression (%)	0.92	1.6	0.28
Periodic paralysis (%)	0.37	0.15	0.35
Pregnancy hypertension (%)	0.92	1.1	0.71
Treatment			
ATC/DDD index of antihypertensive treatment	1.5 (1.0–2.3)	1.0 (0.33–2.0)	< 0.001
Potassium supplement dose (mEq/day)	10.8 (0.0–32.0)	0.0 (0.0–0.0)	< 0.001
Anti-diabetes treatment (%)	9.7	10.0	0.42
Lipid-lowing medication (%)	14.5	15.0	0.44
Screening test			
PAC (pg/mL)	302.0 (206.8–434.5)	156.0 (116.0–213.8)	< 0.001
PRA (ng/mL/h)	0.20 (0.10–0.40)	0.30 (0.20–0.60)	< 0.001
ARR	1232 (661–2321)	435 (285–722)	< 0.001
Confirmatory test: CCT			
PAC on CCT 0 min (pg/mL)	295.0 (191.0–446.0)	133.0 (101.0–180.0)	< 0.001
PRA on CCT 0 min (ng/mL/h)	0.20 (0.10–0.40)	0.30 (0.20–0.50)	< 0.001
ARR on CCT 0 min	1266 (654–2350)	406 (263–653)	< 0.001
PAC on CCT 60 min (pg/mL)	287.5 (187.8–430.0)	101.0 (80.0–135.5)	< 0.001
PRA on CCT 60 min (ng/mL/h)	0.20 (0.10–0.40)	0.30 (0.20–0.60)	< 0.001
ARR on CCT 60 min	1165 (601–2433)	290 (188–488)	< 0.001
PAC on CCT 90 min (pg/mL)	296.0 (186.0–421.0)	103.0 (79.1–138.0)	< 0.001
PRA on CCT 90 min (ng/mL/h)	0.20 (0.10–0.40)	0.40 (0.20–0.60)	< 0.001
ARR on CCT 90 min	1227 (590–2400)	288 (182–475)	< 0.001
Confirmatory test: FUT			
PAC on FUT 0 min (ng/mL/h)	311.0 (208.0–427.8)	130.0 (100.0–178.0)	< 0.001
PRA on FUT 0 min (ng/mL/h)	0.20 (0.10–0.40)	0.30 (0.20–0.50)	< 0.001
PAC on FUT 120 min (ng/mL/h)	501.0 (339.0–697.0)	312.0 (219.0–414.7)	< 0.001
PRA on FUT 120 min (ng/mL/h)	0.40 (0.20–0.80)	0.90 (0.50–1.6)	< 0.001

### Feature importance ranking

**Figure 1:**
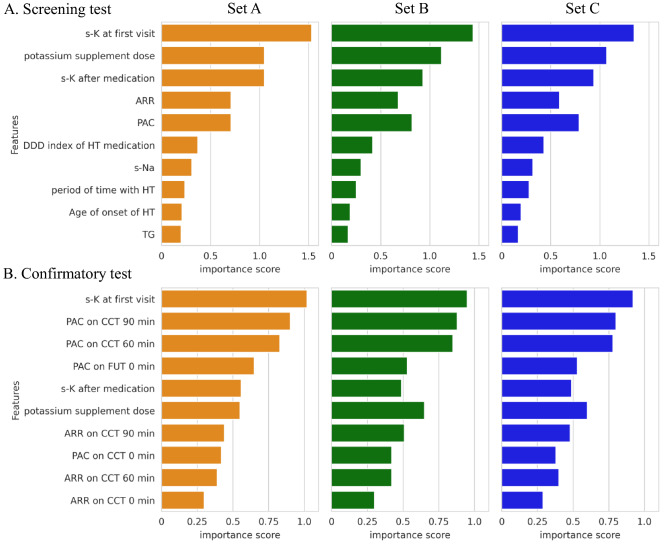
Importance ranking with random forest. (**A**) and (**B**) are rankings in the screening and confirmatory testing models, respectively. There are three different APA datasets depending on the definition of APA. This figure shows 6 of the top-10 importance score rankings. The ranking order is reflected in Set B and Set C with priority given to the order of Set A. *ARR* aldosterone-to-renin ratio, *CCT* captopril-challenge test, *DDD* The ATC/DDD index, *FUT* furosemide-upright test, *HT* hypertension, *PAC* plasma aldosterone concentration, *s-K* serum potassium level, *s-Na* serum sodium level, *TG* triglyceride.

Figure 1 shows the top 10 feature-importance ranking and scores in the RF model of the screening and confirmatory test datasets. The five most important variables of the screening datasets were s-K at first visit, the potassium supplementation dose, s-K after medication, aldosterone-to-renin ratio (ARR), and plasma aldosterone concentration (PAC).

### Performance of MLAs

**Figure 2:**
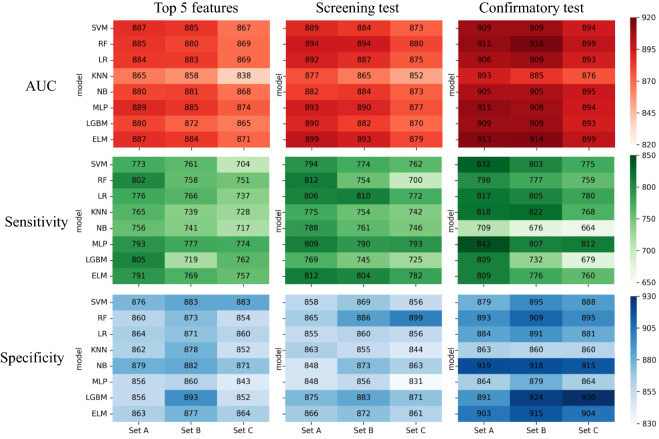
Heatmap comparing the area under the receiver operating characteristic curve, sensitivity, and specificity. The counts in each box are the average of 50 runs and are shown in units of 10^–3^. *AUC* area under the curve, *ELM* ensemble learning model, *KNN* k-nearest neighbor algorithm, *LGBM* light gradient boosting machine, *LR* logistic regression, *MLP* multilayer perceptron, *NB* naïve Bayes, *RF* Random Forest, *SVM* support vector machine.

The combination of the three APA patterns and the three datasets resulted in the development of nine ELM prediction algorithms. Figure 2 shows heatmaps of the performance of the APA prediction models using the screening test dataset, the five most important variables of the screening dataset, and the confirmatory test dataset.

The average AUC of the screening test data set was 0.899 ± 0.043, 0.893 ± 0.041, and 0.879 ± 0.042 for Sets A, B, and C, respectively. In the five most important variable data sets, the model with the highest AUC was 0.889 ± 0.048 (sensitivity 0.793 ± 0.074, specificity 0.856 ± 0.045) for MLP, and the AUC for ELM was 0.887 ± 0.046 (sensitivity 0.791 ± 0.071, specificity 0.863 ± 0.039).

The models with the highest AUCs w”re d’veloped in the confirmatory test data set: 0.913 ± 0.039 (sensitivity 0.809 ± 0.062, specificity 0.903 ± 0.042) for ELM in Set A, 0.916 ± 0.030 (sensitivity 0.777 ± 0.057, specificity 0.909 ± 0.034) for RF in Set B, and 0.899 ± 0.038 (sensitivity 0.760 ± 0.096, specificity 0.904 ± 0.039) for ELM in Set C.

### External validation

**Figure 3:**
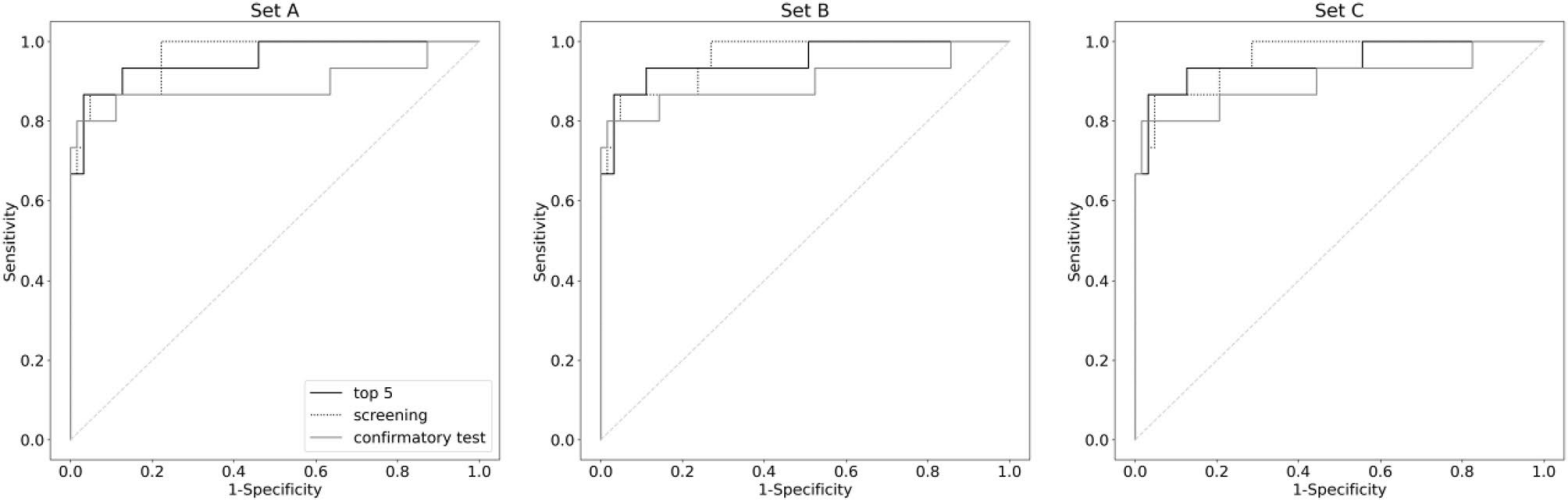
Receiver operating characteristic curves for predictive diagnosis of aldosterone-producing adenoma using external validation data. There are three different APA datasets depending on the definition of APA (Set A, Set B, and Set C). A top 5 model is a model developed using only the top 5 features of the importance score of the screening model. The black line shows the Top 5 model, the dashed line shows the screening model, and the gray line shows the confirmatory test model.

Figure 3 shows the performance of ELMs based on external validation data using the receiver operating characteristic curves (ROC). The AUCs in APA Set A, Set B, and Set C were 0.954, 0.952, and 0.948 for the Top 5 data set; 0.964, 0.960, and 0.959 for the screening data set; and 0.891, 0.897, and 0.899 for the confirmation test data set.

Table 2 shows the sensitivity and specificity for a cut-off value of 0.5 for the APA probability and for the APA probability with the highest Youden index. For all nine ELMs, the sensitivity was improved by using the APA probability with the highest Youden index as the cutoff.

**Table 2:** Comparison of sensitivity and specificity by cutoff value of APA probability in the external validation.

APA definition	Dataset	AUC	Cut-off	Sensitivity	Specificity
Set A	Top 5	0.954	0.50	0.666	0.984
		–	0.21*	0.867	0.968
	Screening	0.964	0.50	0.733	0.984
		–	0.17*	0.867	0.952
	Confirmatory test	0.891	0.50	0.666	1.000
		–	0.39*	0.800	0.984
Set B	Top 5	0.952	0.50	0.666	0.984
		–	0.20*	0.867	0.968
	Screening	0.960	0.50	0.666	0.984
			0.18*	0.867	0.952
	Confirmatory test	0.897	0.50	0.666	1.000
		–	0.36*	0.800	0.984
Set C	Top 5	0.948	0.50	0.666	0.984
		–	0.24*	0.867	0.968
	Screening	0.959	0.50	0.666	0.984
		–	0.21*	0.867	0.952
	Confirmatory test	0.899	0.50	0.666	1.000
		–	0.36*	0.800	0.984

An asterisk indicates the cut-off value when the AUC was highest in the external validation data.

### PA cases predicted with ELM

The APA probabilities obtained by the ELM are shown for two cases already clinically diagnosed with the PA subtype of the disease. The ensemble-learning model (EML) prediction algorithm is repeated 50 times. Table 3 shows the clinical background of the two PA patients: Case 1 was diagnosed as APA by the pathological result, and Case 2 was diagnosed as BAH based on AVS results. Medical information that could not be obtained from the medical records was supplemented in MissForest. Supplementary Table S2 provides detailed clinical information on the two cases. The mean predicted rate of APA for Case 1 ranged from 53.0 to 71.7%. The mean predicted rate of APA for Case 2 ranged from 1.8 to 6.8%.

**Table 3:** Clinical background of APA and BAH cases, and the APA possibility predicted by EML.

PA subtype	Case 1	Case 2
	APA	BAH
Basal information		
Age, Sex	42, M	47, F
BMI (kg/m^2^)	23.5	23.9
Age of onset of HT (years)	39	37
Family history of HT	NA	–
BP at first visit (mmHg)	199/124	160/100
BP after medication (mmHg)	138/78	110/71
Anti-HT medication per day	AML 5 mg DOX 2 mg	AML 5 mg
Top 5 variables		
s-K at first visit (mEq/L)	3.3	4.2
s-K after medication (mEq/L)	3.5	4.0
PAC (pg/mL)	161	98
ARR	322	327
Potassium supplementation dose (mEq/day)	28.8	0
APA probability (%)		
Top 5 variables dataset (set A)	71.7 ± 3.4	6.8 ± 1.2
Top 5 variables dataset (set B)	68.1 ± 4.5	4.2 ± 0.80
Top 5 variables dataset (set C)	70.9 ± 2.9	4.5 ± 0.98
Screening dataset (set A)	71.4 ± 4.0	6.2 ± 1.7
Screening dataset (set B)	68.9 ± 7.3	4.7 ± 1.1
Screening dataset (set C)	66.7 ± 4.8	4.3 ± 0.74
Confirmatory test dataset (set A)	62.7 ± 5.6	2.0 ± 0.44
Confirmatory test dataset (set B)	53.0 ± 9.0	1.8 ± 0.67
Confirmatory test dataset (set C)	54.9 ± 8.4	3.1 ± 0.97

## Discussion

We developed a new prediction algorithm for PA subtype and evaluated its performance. This algorithm differs from conventional PA prediction methods in the following points: (1) The model predicts subtype, not localization; (2) is based on a national multicenter registry database, not a single center; (3) CT findings are not required; (4) provides good predictive performance even with only clinical parameters at screening; and (5) is predictive even in the presence of missing clinical information.

This prediction algorithm should be used by general clinicians, as it provides a more accurate prediction with medical information at screening. As PA is a frequent cause of secondary hypertension, its initial diagnosis is often made by a general practitioner. The Endocrine Society guidelines recommend testing high-risk groups for PA, but unfortunately, these guidelines are not consistently applied by clinicians in practice. As a result, a survey of general practitioners found that PA is dramatically underdiagnosed and undertreated^24–26^. The algorithm’s prediction of two PA cases may be closer to the endocrinologist’s intuition. With the assistance of EML, general clinicians will be able to perform high-quality examinations to avoid missing cases with a high probability of PA. The model using the Set B and C data sets predicts not only the type of disease, but also the benefits of postoperative treatment for patients with APA; thereby indicating the benefits of both surgery and histopathology. Thus, the diagnosis of PA, which is hidden in many hypertension cases, could likely be made more efficiently. It may be a burdensome task for inexperienced clinicians to make decisions based on guidelines. This algorithm may become a reliable partner for non-specialists in PA.

Furthermore, this algorithm can be used even if medical information is deficient or not available. As general clinicians are non-PA specialists, they may neglect to obtain the medical information needed to make a diagnosis. In developing predictive models using machine learning, the method of supplementing missing values with MissForest has been reported to have smaller errors than the method of replacing them with mean or median values^27,28^. Appropriate completion methods for missing values can also maximize large registry data with multiple sites participating.

Our APA prediction algorithm boasts high prediction accuracy even with screening data. Several currently reported scoring-based subtype prediction studies^20–22^ have used single-center enrollment, which may bias PA patients, and included the identification of adrenal tumors on CT as a variable. Kaneko et al. presented a PA-subtype prediction system using four supervised machine learning classifiers in a single-center study^29^. Burrello et al. developed and reported RF-based prediction models for diagnosing the PA subtype using parameters derived from 133 and 82 patients with unilateral and bilateral PA, respectively, in a model comprising six parameters associated with the unilateral PA diagnosis: aldosterone at screening and after confirmatory testing, lowest serum potassium value, presence/absence of nodules, nodule diameter, and CT results^23^. A model for predicting PA subtypes using a combination of mass spectrometer-based steroid profiles and machine learning has also been reported^30^. Other methods (dexamethasone-suppression adrenal scintigraphy^11,31,32^, C-metomidate positron emission tomography-CT^33^, and F-CDP2230^18^ were developed to diagnose unilateral PA^34^, but these are only available in specialized facilities or research centers. By contrast, our algorithm does not require a CT scan, mass spectrometry, or scintigraphy; can be analyzed on a standard computer or smartphone; and can predict PA subtypes in primary care practices.

A confirmatory testing model using captopril-challenge test (CCT) and furosemide-upright test (FUT) results showed better accuracy than a screening model. PA diagnostic guidelines recommend the use of several different loading tests^4,10,11^. However, for some patients, the diagnosis may be confirmed by different loading tests such as the saline infusion test, the oral salt-solution loading test, or the fludrocortisone suppression test. Therefore, PA cases not diagnosed by CCT and FUT cannot be predicted by this algorithm. The fact that the type of confirmatory test does not matter is an advantage of the algorithm using the screening dataset.

External validation showed that screening models tended to be more accurate than confirmatory test models. It is standard practice for artificial intelligence to mathematically predict diagnosis based on whether the predicted probability of APA is higher or lower than 0.5. However, when using the algorithm, one might want to set the cutoff lower than 0.5 to avoid missing subjects with APA. About 0.2 in the screening model and 0.3–0.4 in the confirmatory test model should be the standard to proceed with the test as per the PA diagnostic flowchart.

This study has several limitations. First, PAC in the models has not been measured using a mass spectrometer. Second, this algorithm can indicate the presence of APA, but it cannot determine whether the disease is localized in the left or right adrenal gland. Third, no large-scale prospective study was conducted to evaluate the generalization performance of the EML. Finally, we have not validated the APA probability when this EML is used in patients with essential hypertension (EHT). However, the clinical background of patients with EHT, such as serum potassium and PAC levels, has similar characteristics to BAH compared to APA. Therefore, we expect that EML would correctly answer EHT patients as having a low APA probability. Further investigation is needed in the future.

In summary, we developed a novel PA-subtype diagnosis prediction system combining physical observations, medical history, general laboratory test results, and PA screening data with machine-learning methods. The model answers APA probability with a high degree of accuracy. In the future, ELMs will support the prediction of APA with a high degree of diagnostic accuracy for non-specialists in primary care settings. As a result, it reduces the diagnostic burden on primary care physicians, thereby preventing curable APA patients from falling outside of the PA diagnostic flowchart. Our algorithm is an innovative PA-subtype prediction system that may significantly improve the diagnosis and management of this disease, thereby positively impacting patients and primary care practice.

## Methods

A detailed description of the physical examinations, assay methods, and statistical analysis is available in the Supplementary Appendix.

### Study design and patients

This retrospective cross-sectional analysis was part of the JRAS. The nationwide PA registry in Japan comprised 41 centers—22 university hospitals and 19 city hospitals. The study was performed in accordance with the guidelines for clinical research published by the Japanese Ministry of Health, Labour and Welfare. The study protocol was approved by the Ethics Committee of the National Hospital Organization Kyoto Medical Center (Kyoto, Japan) (the lead center) and the institutional ethics committees of the participating centers with consent waivers. This study was registered with the University Hospital Medical Information Network (UMIN ID: 18756). All procedures were performed in accordance with the 1964 Helsinki Declaration and its later amendments.

Men and women aged 20–90 years who were diagnosed with PA and underwent AVS between January 2006 and December 2018 were enrolled in the JRAS. These patients’ clinical characteristics, biochemical findings, and confirmatory tests results were electronically collected using the online registry. System construction, data security, and registry data maintenance were outsourced to EPS Corporation (Tokyo, Japan) as previously described^35^.

The study was conducted using a dataset that was validated in March 2020. We included patients with data available for clinical characteristics, biochemistry, and AVS. PA was diagnosed according to the Japan Endocrine Society and Japan Society of Hypertension guidelines^11,36,37^. The diagnosis was based on the detection of a PAC (pg/mL) to plasma renin activity (PRA; ng/mL per hour) ratio of > 200 or a PAC to active renin concentration (pg/mL) ratio of > 40, and at least one positive result from the following confirmatory tests: CCT, saline-infusion test, FUT, and oral salt-loading test. Antihypertensive medications were changed to calcium channel or α-adrenergic blockers, which have minimal effects on plasma aldosterone levels (as appropriate) until final diagnosis. The inclusion criteria for this study were a confirmed diagnosis of PA and a successful AVS-based subtype diagnosis. The laterality diagnosis was based on AVS with or without adrenocorticotropic hormone (ACTH; cosyntropin) stimulation. The criterion for successful selective catheterization, the SI, was expressed as the ratio of the cortisol concentration in the adrenal vein to that in the inferior vena cava. Successful catheterization was defined as an SI ≥ 5 with or ≥ 2 without ACTH stimulation^4,38^. Unilateral aldosterone overproduction was confirmed using a lateralized index (LI), derived from the ipsilateral adrenal vein aldosterone-to-cortisol concentration ratio divided by the contralateral aldosterone-to-cortisol ratio. Unilateral PA subtype diagnosis was defined as an LI ≥ 4 with, or ≥ 2 without, ACTH stimulation^4,39^. Complete or partial biochemical improvement was determined 12 months after surgery according to a previous article^40^. The exclusion criteria were cortisol overproduction and failed AVS or partially missing data on AVS (Supplementary Fig. S1). The success or failure of AVS was defined by the selectivity index (SI), as described in the next section. Patients with suspected autonomous cortisol secretion (serum cortisol > 1.8 µg/dL after 1 mg dexamethasone) were excluded^41^.

As an external validation of the developed algorithm, we used data from 78 PA patients (15 APA and 63 BAH) who were not included in the JRAS dataset. This dataset includes 57 and 21 PA patients admitted for AVS at Takaoka City Hospital (Takaoka, Japan) from January 1, 2011 to December 31, 2020 or Kanazawa University Hospital (Kanazawa, Japan) from January 1, 2000 to December 31, 2005, respectively. Additional ethical approvals required for external validation were obtained from both the Ethics Committees of Takaoka Municipal Hospital (receipt number 2–29) and Kanazawa University Hospital (trial number 2012-013).

### Dataset and features

The JRAS database contains the following PA diagnostic criteria: physical observations; medical history; electrolyte, renal function, glucose metabolism, and lipid metabolism parameters; ARR at screening; and confirmatory tests results. Variables obtained at the time of screening tests included 46 items. Variables obtained at the time of confirmatory tests, such as the CCT and the FUT, included 13 items. A maximum of 46 features were available for the screening model, and a maximum of 59 features were available for the confirmatory test model. The handling of datasets and features is described in the Supplementary Appendix.

### Development and performance evaluation of MLAs

**Figure 4:**
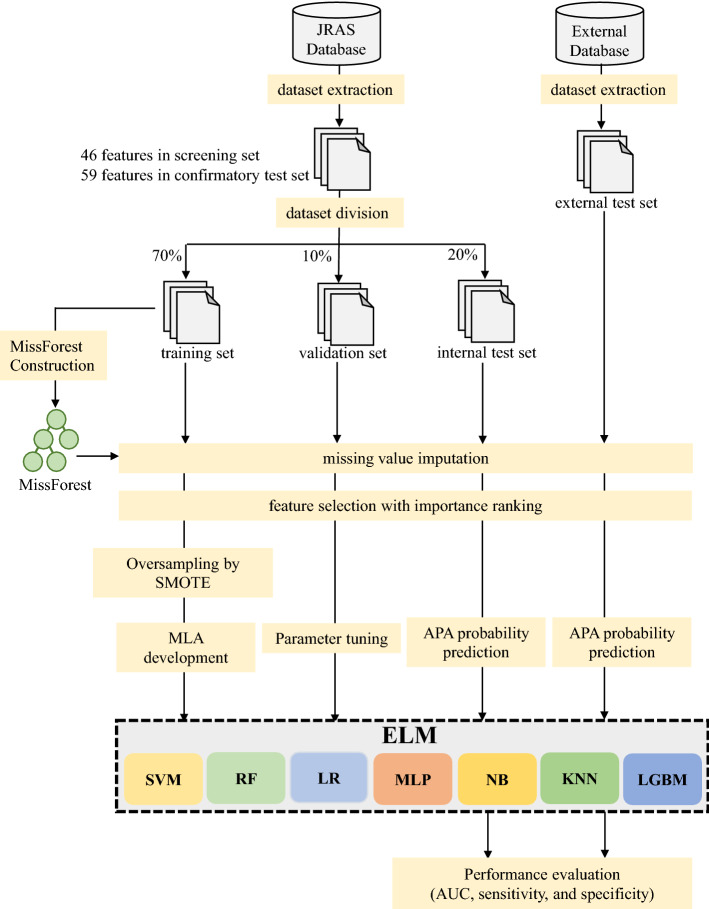
Machine-learning workflow for data processing and model development. MissForest used training data to impute missing values. Feature selection determined the best set of features based on importance ranking analyzed using Random Forest. Oversampling was performed with Synthetic Minority Oversampling Technique (SMOTE) to resolve class imbalance problems. Training, validation, and test datasets were used to train prediction models, parameter tuning, and evaluate generalization performance. The performance of each model was evaluated using the 50-replicate average. Finally, generalization performance was evaluated using an external database. *APA* aldosterone-producing adenoma, *AUC* area under the curve, *ELM* ensemble learning model, *JRAS* Japan Rare/Intractable Adrenal Diseases Study, *KNN* k-nearest neighbor algorithm, *LGBM* Light gradient boosting machine, *LR* logistic regression, *MLA* machine-learning algorithm, *MLP* multilayer perceptron, *NB* naïve Bayes, *RF* Random Forest, *SVM* support vector machine.

The algorithm development process included: (1) data preparation and (2) general process of model construction and evaluation (Fig. 4). Seven machine-learning programs (Random Forest [RF], Multilayer Perceptron [MLP], Light Gradient Boosting Machine [LGBM], Support Vector Machine [SVM], Logistic Regression [LR], k-nearest neighbor algorithm [KNN], and naïve Bayes [NB]) were employed. MLA features were selected to maximize the 50 times average AUC of the model. Each of the seven MLMs outputs the class probability, clinically defined as the ratio of the probability of being a patient with APA. The ELM was then constructed by combining the seven different MLAs. The ELM’s integrated output was calculated by averaging the class probabilities of the individual MLAs. Finally, the generalizability of the ELM was evaluated using data from external PA patients.

### Statistical analyses

The data are expressed as mean ± standard deviation (SD) or percentage. We analyzed the differences between APA and BAH. The Shapiro–Wilk test was used for testing the normality. If normality was rejected, we used the Mann–Whitney *U* test. If normality was accepted, the Bartlett test was used to test equal variance, and Student’s *t*-test or Welch’s t-test was used if equivariance was found or was absent, respectively. *P* < 0.05 was considered statistically significant. We performed the statistical analyses using Python 3.7.7 (Python Software Foundation, Wilmington, Delaware, USA) and SciPy (https://www.scipy.org/scipylib/index.html. Accessed 2020/10/12.) 1.4.1, Scikit-learn 0.22.1^42^.

## Supplementary Information

Supplementary Information.

## Data Availability

The data that support the findings of this study are available from the JPAS/JRAS Study. Restrictions apply to the availability of these data, which were used under license for this study. Data are available from Takashi Yoneda with the permission of the JPAS/JRAS Study.
