# Supplementary material for: A hyperaldosteronism subtypes predictive model using ensemble learning
Source: Sci Rep. 2023 Feb 21;13:3043. doi: 10.1038/s41598-023-29653-2 (PMC9943838; doi:10.1038/s41598-023-29653-2)
Supplement: Supplementary file 1 — Supplementary Information. [file 41598_2023_29653_MOESM1_ESM.docx]

**Supplemental File for Review**

**Supplemental Methods**

1. ***Dataset and features***

The handling of datasets and features is shown in detail. First, we created two datasets from the JRAS database: a screening dataset and confirmatory dataset. The screening dataset contained 46 variables, including age, sex, body mass index (BMI), age at hypertension onset, period of time with hypertension, family history of hypertension, smoking, drinking, systolic (SBP) and diastolic blood pressure (DBP) at first visit, SBP with antihypertensive treatment, DBP with antihypertensive treatment, and daily defined doses (DDD) of antihypertensive agents based on the ATC/DDD index of 2020 (1), a scale that can quantify the type of antihypertensive drug and its potency, and published by the WHO Collaborating Centre for Drug Statistics Methodology annually. Biochemical examination variables were the following: serum potassium level (s-K) at first visit; s-K after medication; daily potassium supplementation dose; serum levels of sodium (s-Na), chloride (s-Cl), creatinine (s-Cr), and uric acid (s-UA); blood urea nitrogen (BUN); estimated glomerular filtration rate (eGFR); urine protein; fasting blood sugar (FBA); HbA1c; total cholesterol (TC); triglycerides (TG); and LDL and HDL cholesterol levels. Endocrinological examination variables were PAC, plasma renin activity (PRA), and ARR. Urine protein was semi-quantitatively examined using five levels: “negative,” “trace,” “1+,” “2+,” and “3+.”

The JRAS database has information related to the medical history and treatment of diseases, such as stroke, ischemic heart disease (IHD), heart failure, atrial fibrillation, chronic kidney disease (CKD), hyperuricemia, diabetes, dyslipidemia, sleep apnea syndrome (SAS), depression, periodic paralysis, and pregnancy-induced hypertension. The diagnostic criteria for these diseases are shown in the Appendix. Additionally, we collected information on therapeutic drugs used in the treatment of diabetes and dyslipidemia as one of the features.

The confirmatory test dataset, which contained 59 features, included the variables of the screening test dataset, and CCT and FUT parameters. Blood samples were collected and PRA, PAC, and ARR were measured before and 60 or 90 min after administering 50 mg of captopril in the CCT, and before and at 120 min in an upright posture after an intravenous bolus injection of 40 mg furosemide in the FUT.

***1.1 Physical examinations***

・The BMI was calculated as weight (kg) divided by height (m) squared.

・The BP was measured by employing an established procedure using a sphygmomanometer with an appropriately sized cuff positioned at the level of the heart (2–4).

***1.2 Biochemistry method***

・The fasting blood sample was obtained early in the morning for the measurement of levels of fasting blood glucose, serum cholesterol, and the other biochemistry parameters, including electrolytes and creatinine levels, which were determined by employing standard laboratory procedures, as described previously (5).

・The glomerular filtration rate was measured using an abbreviated equation, which included serum creatinine (Cre), age, and sex for easy computation: eGFR (mL×min^−1^×1.73 m^−2^)=186×(Cre)^−1.094^×(age)^−0.287^×(0.742 for women) (6).

・Proteinuria was semi-quantitatively diagnosed across 5 levels: “negative”, “trace”, “1+”, “2+” and “3+”.

***1.3 Medical history***

・Stroke included a diagnosis of cerebral infarction, cerebral hemorrhage, and subarachnoid hemorrhage, confirmed by neurologists.

・Ischemic heart disease (IHD) included myocardial infarction (MI) and angina pectoris, which were confirmed by cardiologists; atrial fibrillation was diagnosed based on an electrocardiogram.

・The diagnostic criteria for diabetes were in accordance with the Japan Diabetes Guidelines (7). The diagnostic criteria for dyslipidemia were in accordance with the Japan Atherosclerosis Society Guidelines (8).

・Hyperuricemia, sleep apnea syndrome, depression, periodic paralysis, and pregnancy-induced hypertension data were registered in the JRAS database based on the patient’s self-reported declarations.

***1.4 Treatment information***

・The antihypertensive medications were converted into standardized DDD according to the World Health Organization Anatomical Therapeutic Chemical /DDD Index 2010 (1).

・Daily amounts of potassium (mEq/day) were standardized as daily dose of potassium chloride ; 411 patients were treated for hypokalemia using potassium L-Aspartate (n = 43), [potassium](https://www.weblio.jp/content/Potassium) [glutamate](https://www.weblio.jp/content/glutamate) (n = 5), and potassium chloride (n = 367) among the 1,897 examined patients. We calculated amounts of potassium contained in potassium L-aspartate as being twice that of potassium chloride based on the commonly used dose because of the difference in potassium internalization between potassium L-aspartate and potassium chloride.

***1.5 Assay methods of plasma aldosterone concentration and plasma renin activity***

Plasma aldosterone concentrations were measured using commercially available radioimmunoassays (SPAC-S Aldosterone kits, Fuji Rebio, Co., Ltd, Tokyo, Japan) at 38 centers or Chemiluminescent Enzyme Immunoassay (CLEIA; Accuraseed Aldosterone, FUJIFILM Wako Pure Chemical, Co., Tokyo, Japan) at 3 centers. The PRA was measured using a radioimmunoassay or enzyme immunoassay (EIA). The reference range for PRA with patients in the supine position was 0.3–2.9 ng/mL/hr (PRA-FR RIA kits, Fuji Rebio, Co., Ltd, Tokyo, Japan) at 24 centers, 0.2–2.3 ng/mL/hr (PRA EIA kits, Yamasa, Co., Ltd, Choshi, Japan) at 13 centers, and 0.2–2.7 ng/mL/hr (PRA RIA kits, Yamasa, Co., Ltd, Choshi, 122 Japan) at 3 centers.

1. ***Process to develop the machine-learning algorithms***

***2.1 Data preparation***

For supervised learning, the output variable adopted the PA laterality subtypes: APA as “1” (favorable) and BAH as “0” (unfavorable). Numerical variables were treated as continuous variables. Categorical variables were employed as binary variables, except urinary protein, which comprised five levels.

Input variables included the 46 and 59 variables of the screening and confirmatory test datasets, respectively. The entire process is described in Figure 1. First, manual variable extraction was performed as described in the Dataset and variables subsection. Subsequently, the database was divided into training (70%), validation (10%), and test (20%) datasets, stratified according to the proportion of PA laterality subtypes. The training dataset was used to train prediction models, the validation dataset was used for parameter tuning, and the test dataset was used to measure generalization performance. Lastly, the constructed models were evaluated using area under the receiver operating characteristic curve (AUC), sensitivity, and specificity. Specificity and sensitivity were evaluated using Youden’s index and the AUC. Youden’s index is the point existing at the maximum vertical distance of the receiver operating characteristic curve from the diagonal line and can be calculated by combining the sensitivity and specificity and subtracting 1 (9). The entire process was repeated 50 times, and each model’s performance was evaluated according to the mean value of the replicates.

***2.2 General process of model construction and evaluation***

This study adopted the ensemble learning model (ELM) and employed seven machine learning algorithms (MLA): Random Forest (RF), Multilayer Perceptron (MLP), Light Gradient Boosting Machine (LGBM), Support Vector Machine (SVM), Logistic Regression (LR), k-nearest neighbor algorithm (KNN), and Naive Bayes (NB). The ELM was constructed using multiple different types of MLA and combined the prediction of each model (10–12). The integrated output of the ELM was calculated by averaging the class probability of individual MLAs. The entire ELM was built and compared following five steps. In the first step of model construction, the missing values in each dataset were imputed using MissForest (13), based on training data. Subsequently, we employed feature selection based on feature importance ranking in RF and identified variables using the best-performing prediction models. Each machine-learning model was trained with a feature subset consisting of 1–46 and 1–59 variables in the screening and confirmatory test datasets, respectively. Additionally, a third dataset was developed using the five most important variables according to the importance ranking on RF.

In the third step, Synthetic Minority Oversampling Technique (SMOTE) (14) was used for oversampling until the minority class ratio was equal to the majority class ratio on training data.

All models had hyperparameters that contributed to their performance. The best parameters of the model learned with the training dataset were searched using validation data. In the fourth step, six different algorithms except for NB were optimized for the following parameters in accordance with methods that were previously reported (15–21).

・RF: the number and the depth of trees.

・MLP: the number of hidden layers and neurons, the penalty parameter.

・LGBM: the number and the depth of trees, the regularization strength.

・SVM: the regularization strength and a coefficient for radial basis function (RBF) kernel.

・LR: the regularization strength.

・KNN: the number of nearest neighbors.

All other hyperparameters were set at the default values in Scikit-learn 0.22.1.

In the final step, the prediction models underwent external validation employing a test dataset that had not been utilized in previous steps. These evaluated models were refit by employing the training dataset, the tuned hyperparameters, and the optimized number of features. The ELM’s prediction for the test dataset was calculated by combining the class probability output by the seven MLAs.

Additionally, the generalization performance of each model was evaluated in this step with the measurements of AUC, sensitivity, and specificity.

***2.3 Imputation of missing values using MissForest***

We used MissForest to impute missing value in six algorithms (13). MissForest creates a random forest model for each variable with missing values, using the rest of the variables in the dataset. MissForest then predicts the missing values for that variable. The process is repeated until a terminating criterion is met or the set number of attempts is reached.

***2.4 Oversampling using SMOTE***

We used SMOTE, which is an oversampling technique, to increase the minority class to the same number as the majority class to manage handling of imbalanced data (14). SMOTE generates synthetic data using interpolation with the data detected using the k-neighbor method. The model is constructed in the following steps:

1. Choose a minority sample () and one of k minority neighborhood of as


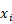

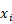

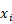

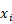

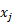

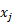


1. Using random number ranged from 0 to 1(gap), difference in attribute values of and (diff)


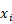

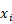

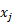

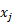


Synthetic data () is


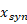

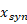

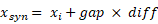


1. Repeat until the same number as the majority class

***2.5 Machine-learning algorithms***

We adopted seven machine-learning algorithms: RF, MLP, LGBM, SVM, LR, KNN, and NB. These algorithms can be described briefly as follows. RF^22^ is a kind of ensemble learning that creates multiple models (called weak learners) and combines them to make a decision. RF comprises decision trees constructed with a different subset of training data, where each tree is slightly different from the others. It can calculate feature importance based on the impurity. MLP^23^ is the principal artificial neural network algorithm. MLP consists of an input layer, a hidden layer, and an output layer with non-linear activation function, and it learns by updating the weights until the error between the output model data and the teacher data is reduced. The advantage of this technique is that more complex network architectures are used to solve more complex problems. LGBM^24^ is a model based on the Gradient Boosting Decision Tree and a kind of ensemble learning. LGBM can build the tree sequentially, which makes it an effective method for processing large-scale data and features that can handle missing values as they are. SVM^25^ constructs a separating decision boundary, called hyperplane, and discriminates by maximizing the margin between classes. The algorithm can map the input feature space to a multi-dimensional space using kernel functions. LR^26^ is a traditional method for binary classification, which is the extension of a linear model. The model is explainable as the effect of the coefficient of the dependent variable on the outcome. KNN^27^ is non-parametric algorithm which performs a classification based on distance metric. The prediction for the test data is assigned by a majority vote of *k* samples that are close in distance to the training data. NB^28, 29^ is a probabilistic model based on the Bayes’ theorem with the strong assumption of conditional independence between input features. An NB classifier is easy to build because it does not require iterative parameter estimation.

***3. References***

1. WHO Collaborating Centre for Drug Statistics Methodology: ATC/DDD Index 2020. Oslo, Norway: Norwegian Institute of Public Health (<https://www.whocc.no/atc_ddd_index/>).
2. Umemura S, Arima H, Arima S, et al. [The Japanese Society of Hypertension Guidelines for the Management of Hypertension (JSH 2019).](https://pubmed.ncbi.nlm.nih.gov/31375757/) Hypertens Res. 2019;42:1235-1481.

# [Shimamoto](https://pubmed.ncbi.nlm.nih.gov/?term=Shimamoto+K&cauthor_id=24705419) K, [Ando](https://pubmed.ncbi.nlm.nih.gov/?term=Ando+K&cauthor_id=24705419) K, [Fujita](https://pubmed.ncbi.nlm.nih.gov/?term=Fujita+T&cauthor_id=24705419) T, et al. The Japanese Society of Hypertension Guidelines for the Management of Hypertension (JSH 2014). Hypertens Res. 2014;37:253-390.

1. [Ogihara](https://pubmed.ncbi.nlm.nih.gov/?term=Ogihara+T&cauthor_id=19300436) T, [Kikuchi](https://pubmed.ncbi.nlm.nih.gov/?term=Kikuchi+K&cauthor_id=19300436) K, [Matsuoka](https://pubmed.ncbi.nlm.nih.gov/?term=Matsuoka+H&cauthor_id=19300436) H, et al. The Japanese Society of Hypertension Guidelines for the Management of Hypertension (JSH 2009). Hypertens Res. 2009;32:3-107.
2. Ohno Y, Sone M, Inagaki N, et al. Prevalence of cardiovascular disease and its risk factors in primary aldosteronism: a multicenter study in Japan. Hypertension. 2018;71:530-537.
3. Matsuo S, Imai E, Horio M, et al. [Revised equations for estimated GFR from serum creatinine in Japan.](https://pubmed.ncbi.nlm.nih.gov/19339088/) Am J Kidney Dis. 2009;53:982-992.
4. Seino Y, Nanjo K, Tajima N, et al. Report of the committee on the classification and diagnostic criteria of diabetes mellitus. J Diabetes Investig. 2010;1:212-228.
5. Teramoto T, Sasaki J, Ishibashi S, et al. Diagnostic criteria for dyslipidemia. J Atheroscler Thromb. 2013;20:655-660.
6. [Hilden](https://pubmed.ncbi.nlm.nih.gov/?term=Hilden+J&cauthor_id=8783436) J, [Glasziou](https://pubmed.ncbi.nlm.nih.gov/?term=Glasziou+P&cauthor_id=8783436) P. Regret graphs, diagnostic uncertainty and Youden's Index. Stat Med. 1996;15:969-986.
7. Alghamdi M, Al-Mallah M, Keteyian S, et al. Predicting diabetes mellitus using SMOTE and ensemble machine learning approach: the Henry Ford ExercIse Testing (FIT) project. PLoS One. 2017;12:e0179805.
8. Lin E, Lin CH, Lane HY. Prediction of functional outcomes of schizophrenia with genetic biomarkers using a bagging ensemble machine learning method with feature selection. Sci Rep. 2021;11:1-8.
9. Park DJ, Park MW, Lee H, Kim YJ, Kim Y, Park YH. Development of machine learning model for diagnostic disease prediction based on laboratory tests. Sci Rep. 2021;11:7567.
10. Stekhoven DJ, Bühlmann P. MissForest—non-parametric missing value imputation for mixed-type data. Bioinformatics. 2012;28:112-118.
11. Chawla N, Bowyer K, Hall L, Kegelmeyer W.: SMOTE: Synthetic Minority Over-Sampling Technique. J Artif Intell Res. 2002;16:321-357.
12. Czarnecki WM, Podlewska S, Bojarski AJ. Robust optimization of SVM hyperparameters in the classification of bioactive compounds. J Cheminform. 2015;7;38.
13. Lee HC, Yoon SB, Yang SM et al. Prediction of acute kidney injury after liver transplantation: machine learning approaches vs. logistic regression model. J. Clin. Med. 2018;7:428.
14. Wong J, Manderson T, Abrahamowicz M, Buckeridge DL, Tamblyn R. Can Hyperparameter Tuning Improve the Performance of a Super Learner?: A Case Study. Epidemiology. 2019;30:521-531.
15. Akiba T, Sano S, Yanase T, Ohta T, Koyama M. Optuna: A next-generation hyperparameter optimization framework. In: KDD '19: Proceedings of the 25th ACM SIGKDD International Conference on Knowledge Discovery & Data Mining. 2019:2623-2631.
16. Probst P, Wright MN, Boulesteix AL. Hyperparameters and tuning strategies for random forest. WIREs Data Mining Knowl Discov. 2019;9:e1301.
17. Zhang J, Mucs D, Norinder U, Svensson, F. LightGBM: an effective and scalable algorithm for prediction of chemical toxicity - application to the Tox21 and mutagenicity datasets. J Chem Inf Model. 2019;59:4150-4158.
18. F Khan, S Kanwal, S Alamri et al. Hyper-Parameter Optimization of Classifiers, Using an Artificial Immune Network and Its Application to Software Bug Prediction. IEEE Access. 2020;8:20954-20964.
19. Breiman L. Random forests. Machine Learning. 2001;45:5-32.
20. Rumelhart DE. Hinton GE, Williams RJ. Learning representations by back-propagating errors. Nature. 1986;323:533-536.
21. Ke G, Meng Q, Finley T, et al. LightGBM: a highly efficient gradient boosting decision tree, in: 31^st^ Conference Neural Information Processing Systems NIPS, 2017;3146-3154.
22. Cortes C, Vapnik V. Support-vector networks. Machine Learning. 1995;20:273-297.
23. DW Hosmer, S Lemeshow, RX Sturdivant. Applied logistic regression 3^rd^ edition. 2013; John Wiley & Sons, Inc.
24. Cover T, Hart P. Nearest neighbor pattern classification. IEEE Trans. Inf. Theory. 1967;13:21-27.
25. Langley P, Iba W, Thompson K. An analysis of Bayesian classifiers. Proceedings of the Tenth National Conference on Artificial Intelligence (AAAI). 1992;223-228.
26. Friedman N, Geiger D, Goldszmidt M. Bayesian network classifiers. Machine Learning. 1997;29:131-163.

**Supplemental material**


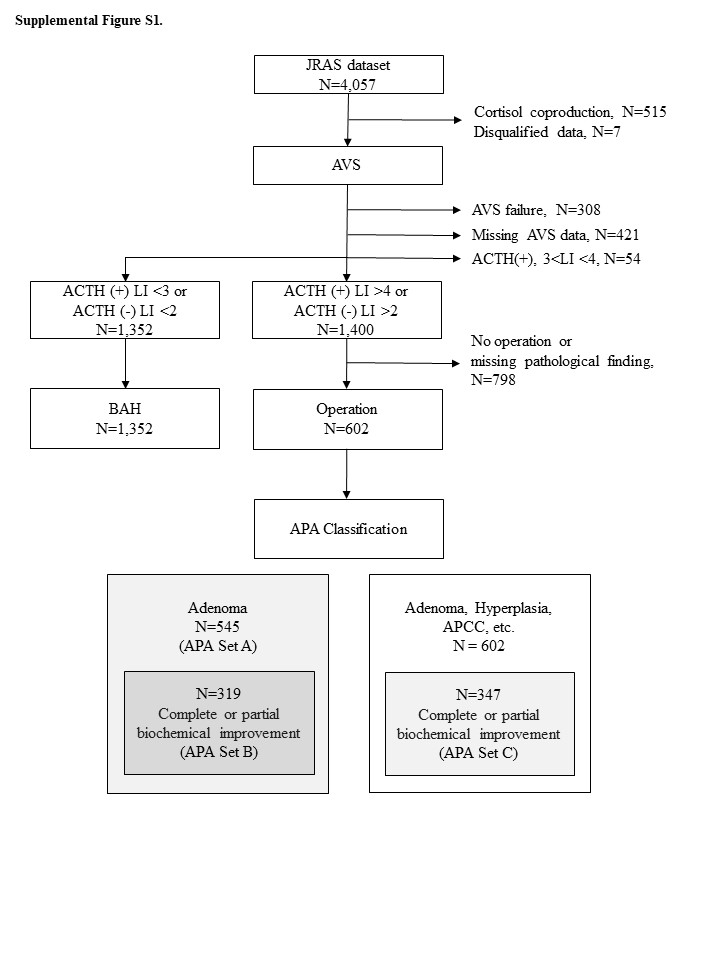


**Supplemental Figure S1. Diagnosis flowchart and Classification definitions for APA datasets.** Abbreviations: ACTH, adrenocorticotropic hormone; APA, aldosterone-producing adenoma; APCC, aldosterone-producing cell cluster; AVS, adrenal venous sampling; BAH, bilateral adrenal hyperplasia; JRAS, Japan Rare/Intractable Adrenal Diseases Study. LI, lateralized index.

| Parameters | Set B  (n=319) | | Set C  (n=347) | |
| --- | --- | --- | --- | --- |
|  |  | P-value |  | P-value |
| Age (years) | 50 (40 - 59) | <0.001 | 49.9 (41 - 59) | <0.001 |
| Sex, Female (%) | 52.4 | 0.50 | 42.3 | 0.09 |
| BMI (kg/m^2^) | 23.6(20.9 - 26.6) | <0.001 | 23.9 (21.0 - 26.7) | <0.001 |
| Age of onset of hypertension (years) | 40 (34 - 48) | <0.001 | 40 (35 - 48) | <0.001 |
| Family history of hypertension (%) | 62.8 | 0.88 | 62.9 | 0.82 |
| Current or past Smoking (%) | 35.9 | 0.53 | 36.2 | 0.59 |
| Current or past Drinking (%) | 54.3 | 0.75 | 55.1 | 0.73 |
| SBP at the first visit (mmHg) | 160 (148 - 180) | <0.001 | 160 (148 - 180) | <0.001 |
| DBP at the first visit (mmHg) | 100 (87- 110) | <0.001 | 100 (88 - 110) | <0.001 |
| SBP with antihypertensive treatment (mmHg) | 140 (129 - 152) | 0.88 | 140 (129 - 153) | 1.00 |
| DBP with antihypertensive treatment (mmHg) | 87 (78 - 96) | 0.50 | 87 (78 - 96) | 0.46 |
| Biochemistry |  | |  | |
| BUN (mg/dL) | 13.0 (10.3- 15.6) | 0.32 | 13.0 (10.4 - 15.6) | 0.40 |
| Serum Creatinine (mg/dL) | 0.71 (0.60 - 0.87) | 0.56 | 0.73 (0.60 - 0.87) | 0.30 |
| eGFR (mL/min per1.73 m^2^) | 79.7 (64.7 – 93.9) | 0.68 | 79.5 (65.0 – 93.7) | 0.62 |
| s-UA (mg/dL) | 5.0 (4.1 - 6.1) | 0.001 | 5.1 (4.2 - 6.2) | 0.007 |
| s-K at the first visit (mEq/L) | 2.9 (2.5 - 3.2) | <0.001 | 2.9 (2.5 - 3.2) | <0.001 |
| s-K with supplement (mEq/L) | 3.3(2.9 - 3.7) | <0.001 | 3.4 (3.0 - 3.7) | <0.001 |
| s-Na (mEq/L) | 143 (141 - 144) | <0.001 | 143 (141 - 144) | <0.001 |
| s-Cl (mEq/L) | 104 (103 - 106) | <0.001 | 105 (103 - 106) | <0.001 |
| FBS (mg/mL) | 96 (89 - 108) | 0.005 | 96 (90 - 108) | 0.02 |
| HbA1c (NGSP) (%) | 5.5 (5.2 - 5.9) | <0.001 | 5.5 (5.2 - 5.9) | <0.001 |
| TC, Total Cholesterol (mg/dL) | 188 (169 - 208) | 0.05 | 188 (169 - 208) | 0.05 |
| TG, Triglyceride (mg/dL) | 95 (65 – 139) | <0.001 | 101 (67 – 143) | <0.001 |
| LDL, LDL-Cholesterol (mg/dL) | 107 (92 - 128) | 0.003 | 108 (92 - 128) | 0.003 |
| HDL, HDL-Cholesterol (mg/dL) | 56 (46 - 67) | 0.18 | 54 (45 - 67) | 0.58 |
| Urine test |  | |  | |
| Urine protein (n, %) |  | |  | |
| (-) or (±) | 244 (83.8) |  | 266 (83.6) |  |
| (+) | 34 (11.7) |  | 38 (12.0) |  |
| (2+) | 6(2.1) |  | 6(1.9) |  |
| (3+) | 7 (2.4) |  | 8 (2.5) |  |
| PA complications |  | |  | |
| Stroke (%) | 5.96 | 0.14 | 6.6 | 0.04 |
| IHD (%) | 2.80 | 0.19 | 3.5 | 0.04 |
| Heart failure (%) | 1.25 | 0.03 | 1.15 | 0.04 |
| Atrial fibrillation (%) | 1.25 | 0.69 | 1.7 | 0.82 |
| CKD (%) | 5.0 | 0.07 | 4.6 | 0.13 |
| Hyperuricemia (%) | 5.6 | 0.06 | 5.8 | 0.04 |
| Diabetes (%) | 12.2 | 0.49 | 12.4 | 0.53 |
| Dyslipidemia (%) | 23.5 | 0.20 | 24.2 | 0.29 |
| SAS (%) | 4.39 | 0.25 | 4.9 | 0.10 |
| Depression (%) | 0.63 | 0.20 | 0.86 | 0.33 |
| Periodic Paralysis (%) | 0.63 | 0.12 | 0.58 | 0.14 |
| Pregnancy hypertension (%) | 0.94 | 0.79 | 0.86 | 0.69 |
| Treatment |  | |  | |
| ATC/DDD index of antihypertensive treatment | 1.5 (1.0 - 2.3) | <0.001 | 1.6 (1.0 - 2.3) | <0.001 |
| Potassium supplement dose (mEq/day) | 16.0 (0.0 - 32.0) | <0.001 | 8.0 (0.0 - 32.0) | <0.001 |
| Anti-diabetes treatment (%) | 9.2 | 0.25 | 9.3 | 0.32 |
| Lipid-lowing medication (%) | 14.9 | 0.49 | 15.2 | 0.61 |
| Screening test |  | |  | |
| PAC (pg/mL) | 315.0 (218.0 – 446.0) | <0.001 | 306.0 (211.0 – 431.0) | <0.001 |
| PRA (ng/mL/hr) | 0.20 (0.20 – 0.40) | <0.001 | 0.25 (0.20 – 0.40) | <0.001 |
| ARR | 1240 (680 - 2341) | <0.001 | 1143 (601 - 2256) | <0.001 |
| Confirmatory test: CCT |  | |  | |
| PAC on CCT 0 min (pg/mL) | 301.5 (197.0 – 453.8) | <0.001 | 238.0 (181.5 - 445.3) | <0.001 |
| PRA on CCT 0 min (ng/mL/hr) | 0.20 (0.10 - 0.40) | <0.001 | 0.20 (0.10 - 0.40) | <0.001 |
| ARR on CCT 0 min | 1374 (680 - 2543) | <0.001 | 1265 (628 - 2350) | <0.001 |
| PAC on CCT 60 min (pg/mL) | 291.0 (178.8 – 430.0) | <0.001 | 272.0 (166.0 – 424.5) | <0.001 |
| PRA on CCT 60 min (ng/mL/hr) | 0.20 (0.10 - 0.40) | 0.006 | 0.20 (0.10 - 0.40) | <0.001 |
| ARR on CCT 60 min | 1250 (600 - 2780) | <0.001 | 1163 (504 - 2609) | <0.001 |
| PAC on CCT 90 min (pg/mL) | 302.0 (188 – 437.5) | <0.001 | 283.5 (167.3 – 423.3) | <0.001 |
| PRA on CCT 90 min (ng/mL/hr) | 0.20 (0.10 - 0.40) | <0.001 | 0.20 (0.10 - 0.40) | <0.001 |
| ARR on CCT 90 min | 1345 (675 - 2500) | <0.001 | 1308 (588 - 2370) | <0.001 |
| Confirmatory test: FUT |  | |  | |
| PAC on FUT 0 min (ng/mL/hr) | 321.0 (208.0 – 454.5) | <0.001 | 307.0 (195.0 - 431.0) | <0.001 |
| PRA on FUT 0 min (ng/mL/hr) | 0.20 (0.10 - 0.40) | 0.05 | 0.20 (0.10 - 0.40) | 0.05 |
| PAC on FUT 120 min (ng/mL/hr) | 540.5 (361.3 – 754.8) | <0.001 | 515.6 (340.3 – 727.5) | <0.001 |
| PRA on FUT 120 min (ng/mL/hr) | 0.40 (0.20 - 0.74) | <0.001 | 0.50 (0.30 - 0.80) | <0.001 |

**Supplemental Table S1.** **Baseline characteristics of patients with APA Set B or Set C.** Data are presented as mean$\pm$SD or percentages. (*P* value; vs BAH) The ATC/DDD index of antihypertensive medication was calculated according to the Anatomical Therapeutic Chemical/Daily Defined Dose Index 2020. Abbreviations: APA, aldosterone-producing adenoma; ARR, aldosterone-to-renin ratio; BAH, bilateral adrenal hyperplasia; BMI, body mass index; BUN, blood urea nitrogen; CCT, captopril-challenge test; CKD, chronic kidney disease; DBP, diastolic blood pressure; eGFR, estimated glomerular filtration rate; FBS, fasting blood sugar; FUT, furosemide-upright test; HbA1c, glycated hemoglobin; HDL, high-density lipoprotein: IHD, ischemic heart disease; LDL, low-density lipoprotein; NGSP, National Glycohemoglobin Standardization Program; PA, primary aldosteronism; PAC, plasma aldosterone concentration; PRA, plasma renin activity; SAS, sleep apnea syndrome; SBP, systolic blood pressure; s-Cl, serum chloride level; s-K, serum potassium level; s-Na, serum sodium level; s-UA, serum uric acid level.

|  | Case 1 | Case 2 |
| --- | --- | --- |
| Basal information |  |  |
| Age (years), Sex | 42, M | 47, F |
| BMI (kg/m^2^) | 23.5 | 23.9 |
| Age of onset of HT (years) | 39 | 37 |
| Family history of HT | NA | - |
| Current or past Smoking / Drinking | - / + | - / - |
| BP at first visit (mmHg) | 199/124 | 160/100 |
| BP after medication (mmHg) | 138/78 | 110/71 |
| anti-HT medication per day | AML 5mg  DOX 2mg | AML 5mg |
| PA Subtype | APA | BAH |
| Biochemistry |  |  |
| BUN (mg/dL) | 12.1 | 11.7 |
| Serum Creatinine (mg/dL) | 0.90 | 0.60 |
| eGFR (mL/min per1.73 m^2^) | 78.3 | 83.0 |
| s-UA (mg/dL) | 3.9 | 3.5 |
| s-K at first visit (mEq/L) | 3.3 | 4.2 |
| s-K after medication (mEq/L) | 3.5 | 4.0 |
| s-Na (mEq/L) | 139 | 139 |
| s-Cl (mEq/L) | 105 | 108 |
| FBS (mg/mL) | 101 | 97 |
| HbA1c (NGSP) (%) | 4.9 | 5.4 |
| Total Cholesterol (mg/dL) | 159 | NA |
| Triglyceride (mg/dL) | NA | 89 |
| LDL-Cholesterol (mg/dL) | 77 | 117 |
| HDL-Cholesterol (mg/dL) | NA | 45 |
| Urine test |  |  |
| Urine protein | - | NA |
| PA complications |  |  |
| Stroke | - | - |
| IHD | - | - |
| Heart failure | - | - |
| Atrial fibrillation | - | - |
| CKD | - | - |
| Hyperuricemia | - | - |
| Diabetes | - | - |
| Dyslipidemia | - | - |
| SAS | + | - |
| Depression | - | - |
| Periodic Paralysis | - | - |
| Pregnancy hypertension | - | + |
| Treatment |  |  |
| ATC/DDD index of antihypertensive treatment | 1.5 | 1.0 |
| potassium supplement dose (mEq/day) | 28.8 | 0.0 |
| Anti-diabetes treatment | - | - |
| Lipid- lowing medication | - | - |
| Screening test |  |  |
| PAC (pg/mL) | 161.0 | 98.0 |
| PRA (ng/mL/hr) | 0.50 | 0.3 |
| ARR | 322 | 327 |
| Confirmatory test: CCT |  |  |
| PAC on CCT 0 min (pg/mL) | 182.0 | 88.3 |
| PRA on CCT 0 min (ng/mL/hr) | 0.20 | 0.10 |
| ARR on CCT 0 min (PRA) | 910 | 883 |
| PAC on CCT 60 min (pg/mL) | 165.0 | 72.6 |
| PRA on CCT 60 min (ng/mL/hr) | 0.40 | 0.20 |
| ARR on CCT 60 min (PRA) | 412.5 | 363 |
| PAC on CCT 90 min (pg/mL) | 199.0 | 67.7 |
| PRA on CCT 90 min (ng/mL/hr) | 0.40 | 0.30 |
| ARR on CCT 90 min (PRA) | 497.5 | 226 |
| Confirmatory test: FUT |  |  |
| PAC on FUT 0 min (ng/mL/hr) | NA | NA |
| PRA on FUT 0 min (ng/mL/hr) | NA | NA |
| PAC on FUT 120 min (ng/mL/hr) | NA | NA |
| PRA on FUT 120 min (ng/mL/hr) | NA | NA |

**Supplemental Table S2. Detailed clinical information on Cases 1 and 2 used for external validation data.** Case 1 and Case2 were predicted by the ELM developed each APA Set A, Set B, and Set C. Variables in parentheses are the variables complemented by MissForest. “NA” is a missing value. Abbreviations: AML, Amlodipine; APA, aldosterone producing adenoma; ARR, aldosterone renin ratio; BAH, bilateral adrenal hyperplasia; BMI, body mass index; BP, blood pressure; BUN, blood urea nitrogen; CCT, captopril-challenge test; CKD, chronic kidney disease; DOX, Doxazosin; eGFR, estimated glomerular filtration rate; FBS, fasting blood sugar; FUT, furosemide-upright test; HbA1c, glycated hemoglobin; HDL, high-density lipoprotein; HT, hypertension; IHD, ischemic heart disease; LDL, low-density lipoprotein; PA, primary aldosteronism; PAC, plasma aldosterone concentration; PRA, plasma renin activity; SAS, sleep apnea syndrome; s-Cl, serum chloride level; s-K, serum potassium level; s-Na, serum sodium level; s-UA, serum uric acid level.
